# Supplementary material for: Characteristics of Physical Exercise Programs for Older Adults in Latin America: A Systematic Review of Randomized Controlled Trials
Source: Int J Environ Res Public Health. 2021 Mar 10;18(6):2812. doi: 10.3390/ijerph18062812 (PMC8000929; doi:10.3390/ijerph18062812)
Supplement: Supplementary file 1 [file ijerph-18-02812-s001.pdf]

Appendix 1. PRISMA 2009 Checklist

Section/topic	#	Checklist item	Reported on page #
**TITLE**			
Title	1	Identify the report as a systematic review, meta-analysis, or both.	1
**ABSTRACT**			
Structured summary	2	Provide a structured summary including, as applicable: background; objectives; data sources; study eligibility criteria, participants, and interventions; study appraisal and synthesis methods; results; limitations; conclusions and implications of key findings; systematic review registration number.	1
**INTRODUCTION**			
Rationale	3	Describe the rationale for the review in the context of what is already known.	2
Objectives	4	Provide an explicit statement of questions being addressed with reference to participants, interventions, comparisons, outcomes, and study design (PICOS).	2
**METHODS**			
Protocol and registration	5	Indicate if a review protocol exists, if and where it can be accessed (e.g., Web address), and, if available, provide registration information including registration number.	PROSPERO 2020 CRD42020208833
Eligibility criteria	6	Specify study characteristics (e.g., PICOS, length of follow-up) and report characteristics (e.g., years considered, language, publication status) used as criteria for eligibility, giving rationale.	3
Information sources	7	Describe all information sources (e.g., databases with dates of coverage, contact with study authors to identify additional studies) in the search and date last searched.	2-3
Search	8	Present full electronic search strategy for at least one database, including any limits used, such that it could be repeated.	Appendix 2
Study selection	9	State the process for selecting studies (i.e., screening, eligibility, included in systematic review, and, if applicable, included in the meta-analysis).	3
Data collection process	10	Describe method of data extraction from reports (e.g., piloted forms, independently, in duplicate) and any processes for obtaining and confirming data from investigators.	3-4
Data items	11	List and define all variables for which data were sought (e.g., PICOS, funding sources) and any assumptions and simplifications made.	3
Risk of bias in individual studies	12	Describe methods used for assessing risk of bias of individual studies (including specification of whether this was done at the study or outcome level), and how this information is to be used in any data synthesis.	4
Summary measures	13	State the principal summary measures (e.g., risk ratio, difference in means).	Not Applied
Synthesis of results	14	Describe the methods of handling data and combining results of studies, if done, including measures of consistency (e.g., I^2) for each meta-analysis.	Not Applied

Appendix 1. PRISMA 2009 Checklist

Section/topic	#	Checklist item	Reported on page #
Risk of bias across studies	15	Specify any assessment of risk of bias that may affect the cumulative evidence (e.g., publication bias, selective reporting within studies).	4
Additional analyses	16	Describe methods of additional analyses (e.g., sensitivity or subgroup analyses, meta-regression), if done, indicating which were pre-specified.	Not Applied
**RESULTS**			
Study selection	17	Give numbers of studies screened, assessed for eligibility, and included in the review, with reasons for exclusions at each stage, ideally with a flow diagram.	5<br>Fig. 1
Study characteristics	18	For each study, present characteristics for which data were extracted (e.g., study size, PICOS, follow-up period) and provide the citations.	5-8
Risk of bias within studies	19	Present data on risk of bias of each study and, if available, any outcome level assessment (see item 12).	9<br>Fig. 2
Results of individual studies	20	For all outcomes considered (benefits or harms), present, for each study: (a) simple summary data for each intervention group (b) effect estimates and confidence intervals, ideally with a forest plot.	9-22
Synthesis of results	21	Present results of each meta-analysis done, including confidence intervals and measures of consistency.	Not Applied
Risk of bias across studies	22	Present results of any assessment of risk of bias across studies (see Item 15).	9<br>Fig. 2
Additional analysis	23	Give results of additional analyses, if done (e.g., sensitivity or subgroup analyses, meta-regression [see Item 16]).	Not Applied
**DISCUSSION**			
Summary of evidence	24	Summarize the main findings including the strength of evidence for each main outcome; consider their relevance to key groups (e.g., healthcare providers, users, and policy makers).	22-24
Limitations	25	Discuss limitations at study and outcome level (e.g., risk of bias), and at review-level (e.g., incomplete retrieval of identified research, reporting bias).	24-25
Conclusions	26	Provide a general interpretation of the results in the context of other evidence, and implications for future research.	25
**FUNDING**			
Funding	27	Describe sources of funding for the systematic review and other support (e.g., supply of data); role of funders for the systematic review.	Not Applied

From: Moher D, Liberati A, Tetzlaff J, Altman DG, The PRISMA Group (2009). Preferred Reporting Items for Systematic Reviews and Meta-Analyses: The PRISMA Statement. PLoS Med 6(6): e1000097. doi:10.1371/journal.pmed1000097

Appendix 2. Search strings for all databases in the systematic review.

**Table 1. Search strings for MEDLINE (by Pubmed)**	
1	(("Exercise"[Mesh]) OR "Exercise therapy"[Mesh]) AND "Aged"[Mesh]
2	FILTER´s:<br>-ARTICLE TYPE: Randomized Controlled Trial<br>-PUBLICATION DAY: 2015 to JUN/2020<br>-AGE: Aged: 65+ years, 80 and over: 80+ years<br>-SPECIES: Humans<br>-LANGUAGE: English, Spanish, Portuguese

**Table 2. Search strings for Elsevier (Scopus)**	
1	(Exercise) OR (Exercise therapy) AND (Aged) AND NOT INDEX (medline)
2	FILTER´s:<br>-Year: 2015 to JUN/2020<br>-Document type: Article<br>-Country/territory: Brazil, Chile, Mexico, Colombia, Argentina, Ecuador, Peru, Cuba, Costa Rica, Uruguay, Venezuela, Jamaica, Puerto Rico, Honduras, El Salvador, Guatemala, Panama, Bolivia<br>-Language: English, Spanish, Portuguese<br>-Subject area: Medicine, Health Professions<br>-Keyword: Aged, Physical Activity, Aging, Resistance Training, Adult, Elderly, Physical Exercise, Training, Strength Training, Very Elderly, Older Adults, Exercise Therapy

**Table 3. Search strings for SciELO**	
1	(Exercise) OR (Exercise therapy) AND (Aged)
2	FILTER´s:<br>-Collection: Brazil, Chile, Colombia, México, Cuba, Argentina, Costa Rica, Perú, Uruguay<br>-Publication Year: 2015 to JUN/2020<br>-Type of Literature: Article<br>-Language: English, Spanish, Portuguese

Appendix 3. Proportions of "Yes" per CERT item for 101 included trials

Item category	CERT item description	N. items rated "Yes"/ N. papers with responses (Percentage)
**WHAT:** materials	C1: Detailed description of exercise equipment (e.g. weights, treadmill, ergometer, etc.)	91/101 90%
**WHO:** provider	C2: Detailed description of instructor expertise, qualifications, and/or training	66/101 65%
**HOW:**	C3: Describe whether exercises are performed individually or in a group	101/101 100%
	C4: Describe whether exercises are supervised or unsupervised; how they are delivered	101/101 100%
	C5: Detailed description of how adherence to exercise is measured and reported	94/101 93%
	C6: Detailed description of motivation strategies	5/101 5%
	C7a: Detailed description of decision rule(s) for determining exercise progression	*5/10 50%
delivery	C7b: Detailed description of how exercise program was progressed	*10/10 100%
	C8: Detailed description of each exercise to enable replication	22/101 21%
	C9: Detailed description of any home program component	*0/0 0%
	C10: Describe any non-exercise components, e.g. education, cognitive behavioral therapy, etc.	*3/3 100%
	C11: Describe the type and number of adverse events that occur during exercise	5/12 41%
**WHERE:** location	C12: Describe the setting in which the exercises are performed	45/101 44%
**WHEN, HOW MUCH:** dosage	C13: Detailed description of exercise intervention, e.g. reps, sets, sessions	91/101 90%
**TAILORING:** what, how	C14a: Describe whether the exercises are generic (one size fits all) or tailored	101/101 100%
	C14b: Detailed description of how exercises are tailored to the individual	58/101 57%
	C15: Describe the decision rule for determining the starting level, e.g. beginner, intermediate, advanced, etc.	4/101 3%
**HOW WELL:** planed, actual	C16a: Describe how adherence or fidelity to the intervention is assessed/measured	94/101 93%
	C16b: Describe the extent to which the intervention was delivered as planned	35/101 34%

* Not all articles carried out this item. Therefore, the total of articles is different from 101.
